# QCM-Based MgFe_2_O_4_@CaAlg Nanocomposite as a Fast Response Nanosensor for Real-Time Detection of Methylene Blue Dye

**DOI:** 10.3390/nano13010097

**Published:** 2022-12-25

**Authors:** Wafa Al-Gethami, Noha Al-Qasmi, Sameh H. Ismail, Ahmed H. Sadek

**Affiliations:** 1 Chemistry Department, Faculty of Science, Taif University, Al-Hawiah, Taif City P.O. Box 11099, Saudi Arabia; 2 Nano Engineering-Xnem Program, Faculty of Nanotechnology for Postgraduate Studies, Sheikh Zayed Campus, Cairo University, 6th October City, Giza 12588, Egypt; 3 Environmental Engineering Program, Zewail City of Science, Technology and Innovation, 6th October City, Giza 12578, Egypt

**Keywords:** sensor, ferrite nanoparticles, alginate nanocomposite, detection, methylene blue, QCM

## Abstract

Methylene blue (MB) dye is a common colorant used in numerous industries, particularly the textile industry. When methylene blue is discharged into water bodies without being properly treated, it may seriously damage aquatic and human life. As a result, a variety of methods have been established to remove dyes from aqueous systems. Thanks to their distinguishing features e.g., rapid responsiveness, cost-effectiveness, potential selectivity, portability, and simplicity, the electrochemical methods provided promising techniques. Considering these aspects, a novel quartz crystal microbalance nanosensors based on green synthesized magnesium ferrite nanoparticles (QCM-Based MgFe_2_O_4_ NPs) and magnesium ferrite nanoparticles coated alginate hydrogel nanocomposite (QCM-Based MgFe_2_O_4_@CaAlg NCs) were designed for real-time detection of high concentrations of MB dye in the aqueous streams at different temperatures. The characterization results of MgFe_2_O_4_ NPs and MgFe_2_O_4_@CaAlg NCs showed that the MgFe_2_O_4_ NPs have synthesized in good crystallinity, spherical shape, and successfully coated by the alginate hydrogel. The performance of the designed QCM-Based MgFe_2_O_4_ NPs and MgFe_2_O_4_@CaAlg NCs nanosensors were examined by the QCM technique, where the developed nanosensors showed great potential for dealing with continuous feed, very small volumes, high concentrations of MB, and providing an instantaneous response. In addition, the alginate coating offered more significant attributes to MgFe_2_O_4_ NPs and enhanced the sensor work toward MB monitoring. The sensitivity of designed nanosensors was evaluated at different MB concentrations (100 mg/L, 400 mg/L, and 800 mg/L), and temperatures (25 °C, 35 °C, and 45 °C). Where a real-time detection of 400 mg/L MB was achieved using the developed sensing platforms at different temperatures within an effective time of about 5 min. The results revealed that increasing the temperature from 25 °C to 45 °C has improved the detection of MB using the MgFe_2_O_4_@CaAlg NCs nanosensor and the MgFe_2_O_4_@CaAlg NCs nanosensor exhibited high sensitivity for different MB concentrations with more efficiency than the MgFe_2_O_4_ NPs nanosensor.

## 1. Introduction

Over the past few decades, nanomaterials with different structures and compositions have been involved in all fields [1,2,3,4]. Among these nanomaterials, magnetic particles have drawn a great deal of attention, and numerous physical experiments have been done on them [5,6,7]. Different uses for magnetic materials were made possible by the ability to create nanoscale magnetic materials [8,9,10]. These numerous applications include ferrofluid technology, electronics, magnetic data storage, magnetocaloric cooling, medication delivery systems, and magnetically targeted contrast agents for magnetic resonance imaging [11].

The chemical formula for ferrites is AB_2_O_4_ (A = Mn, Cu, Mg, Zn, Ni, Co, Cd, and B = Fe). Depending on the characteristics of the divalent cation A, ferrites frequently have a cubic structure resembling a spinel- or inverse-spinel. The trivalent cation B fills the octahedral places in a “typical” spinel structure, while cations A occupies the tetrahedral sites. In the inverse spinel structure, the B cation fills the tetrahedral spaces while an equal number of A and B cations fill the octahedral sites [12,13]. The ferrites that have the formula MgFe_2_O_4_ (which includes magnesium) develop a cubic inverse spinel structure. Due to their extensive applications and exceptional qualities, they have caught the interest of scientists and researchers. Magnesium is an attractive choice for biomedical applications since its magnetic characteristics are softened when used as a divalent cation and because it plays a crucial role in many bodily metabolic processes [14].

MgFe_2_O_4_ nanoparticles have been synthesized using a variety of physical and chemical synthesis methods, including high-energy ball milling, coprecipitation, hydrothermal, solution combustion, and thermal decomposition [15]. The thermal breakdown of organometallic compounds in high-boiling point organic solvents including stabilizing surfactants is one of the most promising wet chemical methods for producing monodisperse magnetic nanoparticles with control over particle size and shape [16]. Magnetic nanoparticles used in environmental applications typically have an inorganic nanoparticle at their core and a biocompatible substance coated on their surface to stabilize them under physiochemical circumstances [17,18,19]. Some nanoparticle systems used in different applications become dependent on these coatings [20]. They simultaneously serve as a steric barrier and, a setting in which the characteristics of the nanoparticles may be modified, preventing the uptake of nanoparticles by macrophages, and reducing the inclination to aggregate [21,22].

The linear anionic polysaccharide alginate, which is mainly composed of the residue of β-(1→4)-linked D-mannuronic acid (M) and α-(1→4)-linked L-guluronic acid (G), is derived from brown seaweed [23]. It can cross-link with divalent cations to create a gel, creating an egg-box shape [24]. For the ionotropic gelation of alginate, calcium is the most popular divalent cation [25]. By slowly dripping the alginate solution into a calcium ion solution, calcium alginate gel (CaAlg) can be generated quickly and easily by extrusion methods [26]. Due to its distinctive gelling properties, CaAlg has been researched as a thickener, stabilizer, and restructuring agent in the food processing industry [27]. In addition, CaAlg has also been employed in cell encapsulation, drug delivery, and tissue engineering [28].

It is generally known that the synthesis process greatly influences the composition, structure, and shape of magnetic ferrite nanoparticles as well as indirectly influences their properties [29]. Biological synthesis is a green method that uses plant, bacteria, and algae extracts for the synthesis of nanomaterials as a straightforward and inexpensive process. Since it doesn’t require risky chemicals, extreme temperatures, or high pressures, it has advantages over other approaches [30,31]. Plant extracts have prospective benefits because of their safety, simplicity of improvement, accessibility, low toxicity, an intricate method of retaining cell structures, and abundance of active agents that can expedite the reduction of metal ions [32]. Other functional components of the plant extract, including polyphenols, amino acids, proteins, terpenoids, ketones, and aldehydes, affect how the nanoparticles are reduced and capped to generate the desired shape and size [33]. The benefit of green synthesis is that it is easy, straightforward, environmentally friendly, non-toxic, and biocompatible. Biological systems such as bacteria, fungi, and plants are considered potential environment-friendly nanofactories. In addition, green synthesis is an alternative to physical and chemical processes due to being non-toxic, affordable, quick, eco-friendly, monodispersed, low in waste output, and able to produce huge quantities. However, there are significant drawbacks to this approach, including difficult-to-control crystal growth, size, shape, aggregation, and stability. Indeed, the possible presence of endotoxin, and time-consuming purification processes [34]. The clove (*Syzygium aromaticum* L.) is a fragrant flower bud of an Indonesian plant and due to it being a significant source of phenolic compounds, thus it is one of the best options for biological activities [35].

The quartz crystal microbalance (QCM) sensor is a simple, highly adjustable, and sensitive sensor for monitoring activity on surfaces or even within thin films [36]. The mass of any target analyte is a fundamental property, and QCM systems could identify these analytes based on their mass, therefore labels are not necessary [37]. Analyses of environmental, food, and biomedical media are frequently conducted using the QCM [38]. Owing to the complexity of these substances, it is imperative to create specific methods for identifying target analytes within them. Since QCM sensors are neither selective nor specific, thus usage of QCM sensors in some applications is thereby becoming challenging [39].

Dye usage is widespread in the textile, paper, pharmaceutical, leather, and plastic industries [40,41]. Once large amounts of dye are dumped into wastewater on a large scale, it may endanger aquatic life, ecosystems, and human health if it enters the food chain [42]. Due to their potential for disruption and poor environmental degradation, many governments have restricted the use of some dyes or discharged them into aquaculture regions [43,44]. One of the pigments that are most frequently used is methylene blue (MB). The MB is considerably used as a model dye to evaluate how well nanocomposites bind to surfaces [45,46]. The ion exchange, electrostatic interactions, or π-π interactions have all been proven to be effective ways for MgFe_2_O_4_ nanoparticle-based nanocomposites to adsorb both cationic and anionic dyes [47]. This implies that MB could be selectively detected using MgFe_2_O_4_ nanoparticles-based alginate nanocomposites.

In this study, QCM-Based MgFe_2_O_4_ NPs and MgFe_2_O_4_@CaAlg NCs Nanosensors were used to fabricate a novel detection technique for MB. First, the magnesium ferrite nanoparticles (MgFe_2_O_4_ NPs) were prepared using clove extract as a green synthesis approach method, and then the synthesized nanoparticles the cross-linked with alginate hydrogel to obtain magnesium ferrite-coated calcium alginate nanocomposite (MgFe_2_O_4_@CaAlg NCs). Both MgFe_2_O_4_ NPs and MgFe_2_O_4_@CaAlg NCs subjected to many characterization tools e.g., X-ray diffraction (XRD), dynamic light scattering (DLS), zeta sizer analysis (ζ-potential), and transmission electron microscopy (TEM) to provide information about the crystallinity, particle size distribution, surface charge, and shape of the prepared samples. Afterward, the synthesized MgFe_2_O_4_ NPs and MgFe_2_O_4_@CaAlg NCs were employed as nanosensor materials based on the QCM technology to real-time monitor the high concentrations of MB dye in the aqueous solutions at different solutions temperatures.

## 2. Materials and Methods

### 2.1. Materials

Iron(III) chloride hexahydrate (FeCl_3_·6H_2_O, ACS reagent, 97%), Magnesium(II) chloride hexahydrate (MgCl_2_·6H_2_O, ACS reagent, 99%), sodium hydroxide (NaOH reagent grade, ≥98%, pellets (anhydrous)) were used to prepare MgFe_2_O_4_ NPs, where sodium alginate (C_6_H_9_NaO_7_, molecular weight of 216.12 g/mol, viscosity 5.0–40.0 cps (c = 1%, H_2_O @ 25 °C)), calcium chloride (CaCl_2_, anhydrous, granular, ≤7.0 mm, ≥93.0%) were used to coat the synthesized MgFe_2_O_4_ NPs by alginate hydrogel to obtain MgFe_2_O_4_@CaAlg NCs. Methylene blue dye (C.I. 52015) (C_16_H_18_ClN_3_S·xH_2_O, a molecular weight of 319.85 g/mol, Reag. Ph Eur) was used as a model contaminant. All chemicals were purchased from Sigma-Aldrich (Munich, Germany) and used as received without any more purification. All solutions were prepared using double-distilled water throughout this work.

### 2.2. Preparation of the Clove Leaves Extract

The clove (*Syzygium aromaticum* L.) leaves were initially cleaned with tap water. Then, they were rinsed with double-distilled water to eliminate impurities and contaminants. After that, they were given five days to dry naturally and 6 g of dried leaves were combined with 100 mL of double-distilled water to yield the leaf extract. Then they were heated for 30 min at 60 °C. The clove leaf extract was then filtered and preserved for future work [48].

### 2.3. Green Synthesis of MgFe_2_O_4_ Nanoparticles

Spinel MgFe_2_O_4_ NPs were prepared using the co-precipitation technique with a few minor modifications and 5 g of FeCl_3_·6H_2_O and 3 g of MgCl_2_·6H_2_O were dissolved in double-distilled water. The mixture was then heated at 50 °C on a hot plate for approximately 15 min. The chloride solution was then vigorously stirred before adding 10 mL of the clove leaf extract. The pH of the mixture was increased to 10 by a few drops of a 0.5 M NaOH solution. As the pH of the solution turned to pH 10, a brown precipitate was formed and allowed to settle down. Thereafter, the mixture was agitated for 2 h at 60 °C. After being rinsed with double-distilled water, the obtained nanoparticles were calcined for 2 h at 600 °C.

### 2.4. Preparation of MgFe_2_O_4_@CaAlg Nanocomposite

Ionotropic gelation was used to prepare the MgFe_2_O_4_@CaAlg NCs. In 50 mL of distilled water, 0.025 g of MgFe_2_O_4_ NPs was sonicated for 20 min. Then, the MgFe_2_O_4_ NPs solution received 1 g of sodium alginate, which was agitated for 1 h before being sonicated for 20 min. Subsequently, a 2 M of calcium chloride solution was added to the mixture, and it was agitated for 1 h. The mixture was refrigerated for two days. The produced nanocomposite was filtered and rinsed three times using distilled water. Following preparation, the MgFe_2_O_4_@CaAlg NCs were dried in an oven for 2 days at 60 °C.

### 2.5. Instrumentation

X-ray diffraction (EQUINOX 1000, Thermo Scientific CO., Lafayette, CO, USA) was used to determine the composition and phase of both green-synthesized MgFe_2_O_4_ NPs and MgFe_2_O_4_@CaAlg NCs. Cu Kα radiation with a current of 31 mA and an applied voltage of 33 kV was used. The 2θ angles ranged between 0° to 85°, and the scan speed was adjusted to 0.1°/min. In addition, the surface charge and particle size of the greenly synthesized MgFe_2_O_4_ NPs and MgFe_2_O_4_@CaAlg NCs were determined using the zeta seizer instrument (NanoSight NS500, Malvern Panalytical, Malvern, UK). The prepared samples were further examined with a TEM instrument (JEOL, JEM-2100 high-resolution, Peabody, MA, USA) to determine the morphology of green-synthesized MgFe_2_O_4_ NPs and MgFe_2_O_4_@CaAlg NCs. The MgFe_2_O_4_ NPs and MgFe_2_O_4_@CaAlg NCs were sonicated for 20 min using an ultrasonic probe sonicator (UP400S, Hielscher, Oderstraße, Teltow, Germany) at a frequency of 55 kHz, an amplitude of 55%, and a cycle of 0.55 before TEM analysis. The dispersed mixture was then deposited in drops with a diameter of five to ten microns across a copper grid that had been coated with carbon before being subjected to TEM analysis.

### 2.6. Establishing of QCM-Based MgFe_2_O_4_ NPs and MgFe_2_O_4_@CaAlg NCs Nanosensors

The QCM sensor is contained an AT-cut quartz crystal chip attached to a gold electrode with a diameter of 12 mm, and a resonance frequency of 5 MHz (Q-Sense, Shenzhen, China). Prior to the stabilization of the nanomaterials, the gold sensor was cleaned by immersing it in a 5:1:1 v/v/v solution of aqueous ammonia, H_2_O_2_, and double-distilled water for 10 min at 75 °C. Then, the gold sensor was rinsed with double-distilled water, and ethanol, and allowed to dry at room temperature. The dried chip was subsequently inserted into the Q-Sense instrument. Afterward, a stream of double-distilled water was first injected over the electrode to serve as a background electrolyte. Injecting the background electrolyte solution (double distilled water) into the QCM module enables the baseline measurements before adding the sensor’s nanomaterials. In order to keep the QCM signal steady, the QCM module was continuously fed by double-distilled water until the value of the QCM signal was then recorded as zero. Then, 2 mL of 25 g/L aliquots of the MgFe_2_O_4_ NPs or MgFe_2_O_4_@CaAlg NCs were dispersed in 10 mL of double distilled water. Following that, aliquots of the mixture were flushed on the gold sensor at a flow rate of 0.1 mL/min.

### 2.7. QCM-Monitoring of MB Dye

The QCM measurements were carried out using a QCM system (QCM, Q-senses, Biolin Scientific, Linthicum Heights, MD, USA). Each QCM measurement was performed by injecting 400 mg of MB solutions (2 mL of 10 g/L MB was dissolved in 50 mL of double distilled water) onto the surface of either QCM-based MgFe_2_O_4_ NPs or MgFe_2_O_4_@CaAlg NCs nanosensors at various temperatures (25 °C, 35 °C, and 45 °C). The MB solution was then injected repeatedly until the signal stabilized, indicating that the equilibrium of the binding interaction between the nanosensors and the MB had been reached. To clean unadsorbed MB off the surfaces of the QCM sensors, double distilled water was once more poured into the module after a predetermined certain time.

## 3. Results and Discussion

### 3.1. Characterization of Green Synthesized MgFe_2_O_4_ NPs and MgFe_2_O_4_@CaAlg NCs

#### 3.1.1. XRD

**Figure 1:**
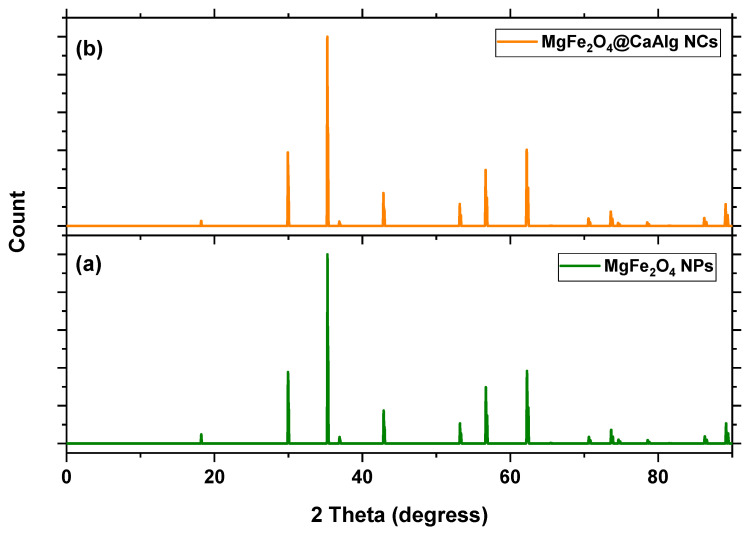
The XRD patterns of (**a**) green synthesized MgFe_2_O_4_ NPs and (**b**) MgFe_2_O_4_@CaAlg NCs, respectively.

The XRD patterns of green-synthesized MgFe_2_O_4_ NPs and MgFe_2_O_4_@CaAlg NCs are shown in Figure 1a and Figure 1b, respectively. The identical 2θ values can be seen at the (111), (220), (311), (400), (422), (511), (440), (533), (622), and (444) planes for MgFe_2_O_4_ NPs proving that MgFe_2_O_4_ has a cubic spinel structure. Sharp, narrow, and strong diffraction peaks demonstrated the high crystallinity of the green-synthesized MgFe_2_O_4_ NPs. No impurity peaks could be seen in both diffractograms except some minor differences were observed in the pattern of MgFe_2_O_4_@CaAlg NCs, which revealed the efficient coating of MgFe_2_O_4_ NPs by Ca-Alginate hydrogel.

#### 3.1.2. DLS and Zeta Potential

**Figure 2:**
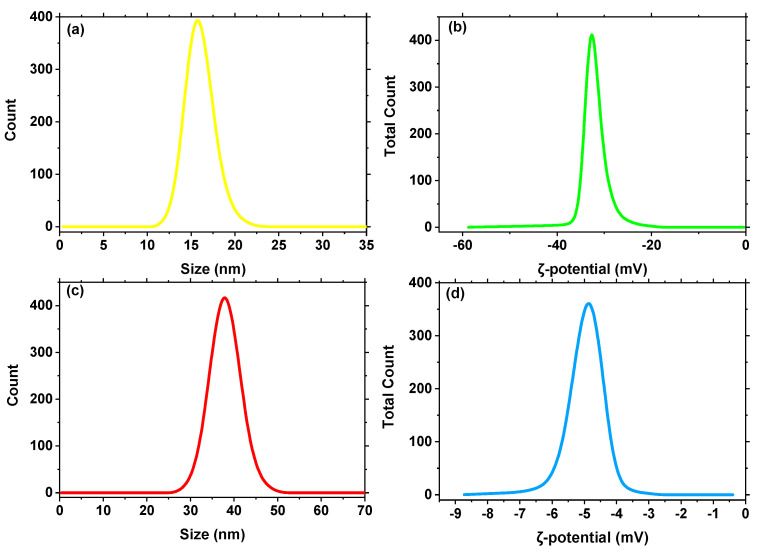
(**a**,**c**) Particle size distribution of green synthesized MgFe_2_O_4_ NPs (yellow line) and MgFe_2_O_4_@CaAlg NCs (red line), (**b**,**d**) surface charge of green synthesized MgFe_2_O_4_ NPs (green line) and MgFe_2_O_4_@CaAlg NCs (blue line), respectively.

The DLS method was used to determine the particle size of green-synthesized MgFe_2_O_4_ NPs and MgFe_2_O_4_@CaAlg NCs (Figure 2a,c). The green-synthesized MgFe_2_O_4_ NPs and MgFe_2_O_4_@CaAlg NCs were found to have average sizes of 15 and 37 nm, respectively. The results showed that all the suspensions had a unimodal size distribution with polydispersity indices and that they all had a high level of colloidal stability. The observed increases in the average size of the MgFe_2_O_4_@CaAlg NCs demonstrate the effective stabilizing of MgFe_2_O_4_ NPs with alginate hydrogel. The average size also considers the existence of nanoparticles and any solvent molecules that are connected to the tumbling particle as a hydrodynamic size.

The stability of green-synthesized MgFe_2_O_4_ NPs and MgFe_2_O_4_@CaAlg NCs in aqueous environments was examined by measuring the ζ-potentials of these materials at different applied voltage values. The measured ζ-values for green-synthesized MgFe_2_O_4_ NPs and MgFe_2_O_4_@CaAlg NCs were −32 and −4.8 mV, respectively, as shown in Figure 2b,d. The ζ-value of MgFe_2_O_4_@CaAlg NCs was decreased negatively, which may be due to masking alginate structures for the negative charge of MgFe_2_O_4_ NPs. This implies the good linking between the CaAlg hydrogel and MgFe_2_O_4_ NPs.

#### 3.1.3. TEM

**Figure 3:**
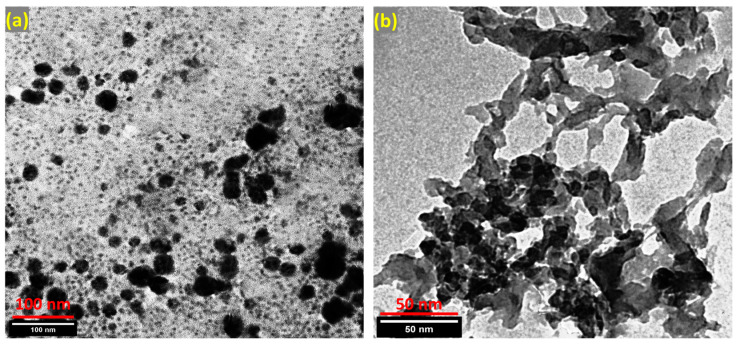
(**a**,**b**) TEM images of the green synthesized MgFe_2_O_4_ NPs and MgFe_2_O_4_@CaAlg NCs, respectively.

The TEM image of green-synthesized MgFe_2_O_4_ NPs further demonstrated the dispersity of the synthesized particles, where the individual particles formed in regular spherical structures with diameters smaller than 100 nm (Figure 3a). At the same time, the image of the MgFe_2_O_4_@CaAlg NCs revealed that the MgFe_2_O_4_ NPs are also evidently coupled to the alginate network. Despite the alginate coating causing the particles’ size to grow, they are still smaller than 100 nm (Figure 3b).

### 3.2. MB Monitoring Using QCM-Based MgFe_2_O_4_ NPs and MgFe_2_O_4_@CaAlg NCs Nanosensors

**Figure 4:**
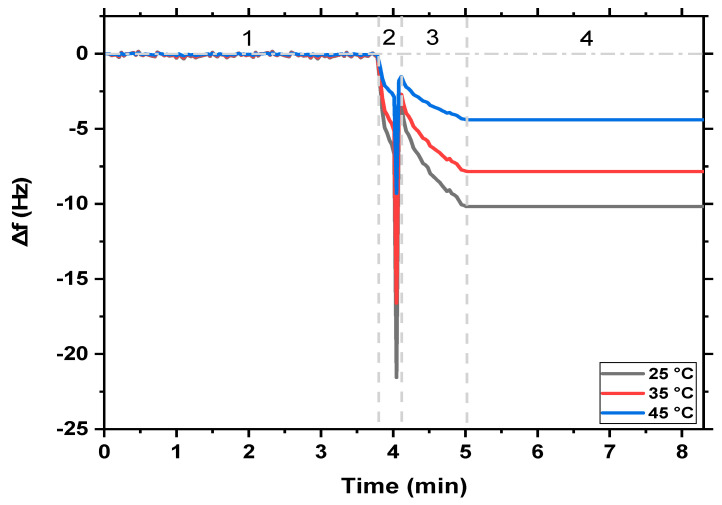
The real-time detection of MB dye (400 mg/L) by MgFe_2_O_4_ NPs nanosensor at different temperatures (25 °C, 35 °C, and 45 °C); zone (1) shows the QCM monitoring of MgFe_2_O_4_ NPs nanosensor, (2) indicates the first injection of MB, (3) represents the adsorption of MB on the nanosensor surface, and (4) adsorption equilibrium of MB.

**Figure 5:**
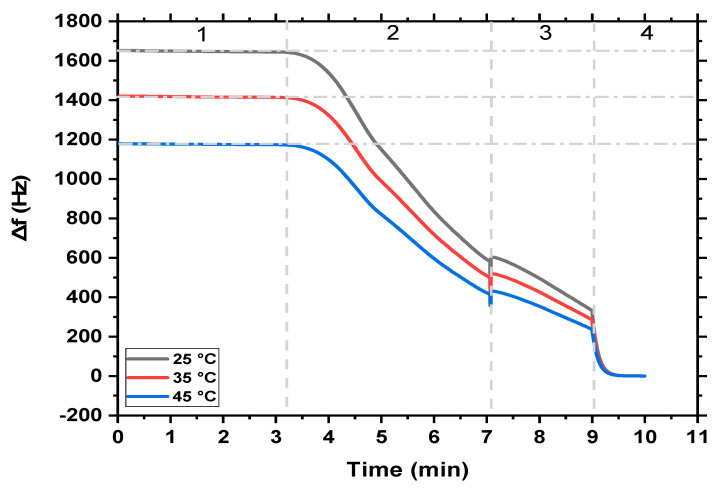
The real-time detection of MB dye (400 mg/L) by MgFe_2_O_4_@CaAlg NCs nanosensor at different temperatures (25 °C, 35 °C, and 45 °C); zone (1) shows the QCM monitoring of MgFe_2_O_4_@CaAlg NCs nanosensor, (2) indicates the monitoring of MB, (3) represents the adsorption of MB on the nanosensor surface, and (4) adsorption equilibrium of MB.

The influence of temperature on the monitoring of the high concentration of MB was achieved at 25 °C, 35 °C, and 45 °C using MgFe_2_O_4_ NPs and MgFe_2_O_4_@CaAlg NCs nanosensors. Figure 4 and Figure 5 revealed that the detection sensitivity of the MB in the aqueous solutions is affected by the medium temperature. A typical QCM-based MgFe_2_O_4_ NPs and MgFe_2_O_4_@CaAlg NCs nanosensors experiments have four stages, (1) represents the frequency response of MgFe_2_O_4_ NPs and MgFe_2_O_4_@CaAlg NCs nanosensors (stable baseline), (2) suddenly drop in the frequency change due to the rapid binding of MB molecules with the sensors, which may be attributed to the large numbers of vacant sites on the sensors’ surfaces, (3) more adsorption of target MB molecules, and (4) equilibrium state of adsorption process between the MgFe_2_O_4_ NPs and MgFe_2_O_4_@CaAlg NCs nanosensors and MB molecules. The frequency shift would stay steady after adding the MgFe_2_O_4_ NPs and MgFe_2_O_4_@CaAlg NCs before the MB solution was pumped into the QCM system. The frequency would drastically change when MB was adsorbed on the surface of QCM-based MgFe_2_O_4_ NPs and MgFe_2_O_4_@CaAlg NCs nanosensors. According to Figure 4 and Figure 5, the frequency was more shifted for the QCM-based MgFe_2_O_4_@CaAlg NCs nanosensor than for the QCM-based MgFe_2_O_4_ NPs nanosensor. This suggests that the QCM-based MgFe_2_O_4_@CaAlg NCs nanosensor was more capable of binding MB molecules because it had more vacant cavities as a result of the presence of the alginate hydrogel. Similar changes in the frequencies of both QCM-based MgFe_2_O_4_ NPs and MgFe_2_O_4_@CaAlg NCs nanosensors have occurred, therefore, indicating that both nanosensors have comparable responses toward the adsorption of MB molecules. Once the frequency became stable again, this means an equilibrium state of MB adsorption on the surface of both QCM-based MgFe_2_O_4_ NPs and MgFe_2_O_4_@CaAlg NCs nanosensors has reached. At step four, no noticeable changes in the frequency of sensors were observed, indicating that minimal mass was lost, and the nanosensor surfaces had only minor structural modifications. This indicated that the QCM-based MgFe_2_O_4_ NPs and MgFe_2_O_4_@CaAlg NCs nanosensors could be used effectively for the real-time detection of MB dye even at high concentrations reaching 400 mg/L. Where chemical reactions are known to be significantly influenced by temperature; hence, a particular reaction may be improved or inhibited by temperature depending on the surroundings of the reactants and/or products. The adsorbate molecule diffuses more quickly through the adsorbent’s exterior boundary layer and inside its pores because of the temperature change. Additionally, changing the temperature will enhance the adsorbent’s capacity to reach equilibrium for a given adsorbate [49].

For adsorption of MB by QCM-based MgFe_2_O_4_ NPs and MgFe_2_O_4_@CaAlg NCs nanosensors, the same adsorption behavior was observed at the different temperatures, but in the case of QCM-based MgFe_2_O_4_ NPs nanosensor, it was found that as the temperature increases from 25 °C to 45 °C, a decrease in the frequency shifts occurred contrary to what happens in the case of QCM-based MgFe_2_O_4_@CaAlg NCs nanosensor, where the frequency shifts were decreased descending. This may be attributed to the fact that the binding between QCM-based MgFe_2_O_4_ NPs nanosensor and cationic MB molecules is based on the electrostatic attraction between the highly negatively charged surface of QCM-based MgFe_2_O_4_ NPs nanosensor and the positively charged MB molecules. Therefore, the temperature increases may cause more diffusion of MB molecules inside the solution and reduce the attaching of MB dye to the surface of the QCM-based MgFe_2_O_4_ NPs nanosensor. Additionally, the bond splitting of the reactive groups on the surface of the QCM-based MgFe_2_O_4_ NPs nanosensor at a higher temperature may cause a reduction in the number of active adsorption sites, which also led to a decrease in the MB’s adsorption amplitude. While in the case of the MgFe_2_O_4_@CaAlg NCs nanosensor, the temperature increases caused physical changes in the alginate hydrogel structure such as rupture strength increases, which may help in the easy diffusion of MB molecules into the surface of the MgFe_2_O_4_@CaAlg NCs nanosensor due to increasing the swelling degree of alginate hydrogel. In addition, this improvement in MB detection may be attributed to the increase of the mobility of MB molecules and the allowed number of active spots on the surface of the MgFe_2_O_4_@CaAlg NCs nanosensor.

**Figure 6:**
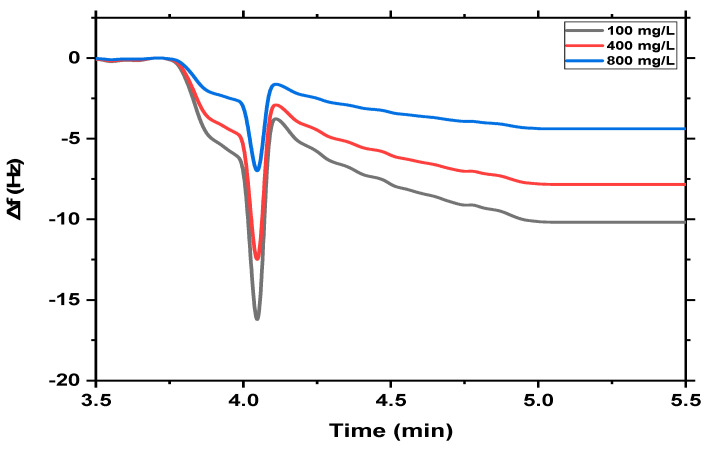
The real-time detection of different concentrations of MB dye (100 mg/L, 400 mg/L, and 800 mg/L) on the surface of the MgFe_2_O_4_ NPs nanosensor at room temperature.

**Figure 7:**
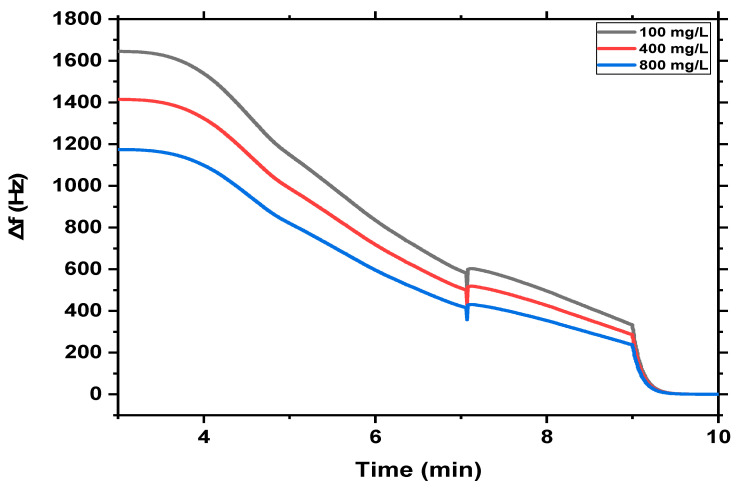
Illustrates the real-time detection of different concentrations of MB dye (100 mg/L, 400 mg/L, and 800 mg/L) on the surface of the MgFe_2_O_4_@CaAlg NCs nanosensor at room temperature.

The sensitivity of both QCM-based MgFe_2_O_4_ NPs and MgFe_2_O_4_@CaAlg NCs nanosensors towards the different concentrations of MB (100 mg/L, 400 mg/L, and 800 mg/L) was investigated as shown in Figure 6 and Figure 7. It could be observed that the adsorption of MB on the surface of the QCM-based MgFe_2_O_4_ NPs nanosensor causes the frequency to change markedly because of the mass of MB that adsorbed onto the sensor surfaces. However, the change in frequency of the QCM-based MgFe_2_O_4_ NPs nanosensor decreased gradually as the MB concentration increased (Figure 6). This may be attributed to the relatively high surface area and the availability of adsorption sites at low initial solution concentrations, where the MB was easily adsorbed and detected. While at higher initial solution concentrations, a decrease in the frequency response of MB occurs due to the limitation or saturation of total available adsorption sites on the surface of the QCM-based MgFe_2_O_4_ NPs nanosensor. This may be due to the formation of monolayer coverage at the interface of the MgFe_2_O_4_ NPs. In addition, this behavior could be attributed to the accumulation of more amount of MB molecules at higher concentrations. Accordingly, the increasing total accumulation of methylene blue is probably due to more contact of QCM-based MgFe_2_O_4_ NPs nanosensor sites with MB molecules. Most of the methylene blue in the sample solution may interact with the adsorbent’s active sites at low concentrations, but as the concentration is increased, more and more methylene blue species will be unavailable to interact with the active surface because the active sites have already been occupied. Moreover, the accumulation of MB molecules results in increasing the positive charge on the surface of QCM-based MgFe_2_O_4_ NPs and causes a raising of repulsion force between MB molecules. Therefore, it could conclude that the sensitivity of QCM-based MgFe_2_O_4_ NPs nanosensors decreases at higher concentrations of MB.

On the other hand, in the case of QCM-based MgFe_2_O_4_@CaAlg NCs nanosensor, the changes in the frequency of the sensor are directly related to the concentration of MB. Where increasing MB concentration from 100 mg/L to 800 mg/L led to a direct increase in the mass loaded on the surface of QCM-based MgFe_2_O_4_@CaAlg NCs nanosensor and resulted in frequency changes in a similar manner where the adsorption capacity of QCM-based MgFe_2_O_4_@CaAlg NCs nanosensor increased with increasing initial MB concentration. This reflects the role of alginate hydrogel in providing more available active sites and further capacity for more MB detection at high concentrations. Therefore, the high sensitivity efficiency of QCM-based MgFe_2_O_4_@CaAlg NCs nanosensor at these concentrations indicated the potential application of QCM-based MgFe_2_O_4_@CaAlg NCs nanosensor for the detection of a high amount of MB.

### 3.3. Proposed Sensing Mechanism of the QCM-Based MgFe_2_O_4_ NPs and MgFe_2_O_4_@CaAlg NCs Nanosensors

**Figure 8:**
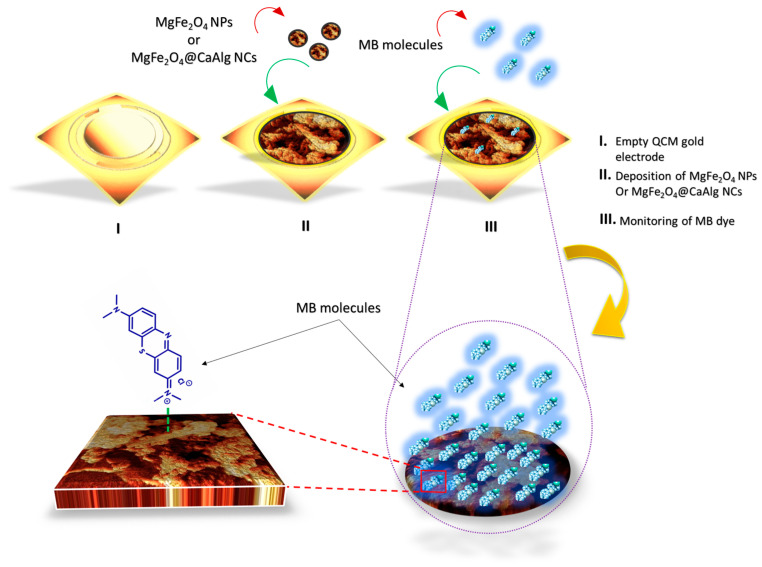
Illustrates the schematic exemplification for the hypothesized interaction mechanism between the QCM-based MgFe_2_O_4_ NPs or MgFe_2_O_4_@CaAlg NCs nanosensors and MB molecules.

The receiving of MB by the QCM-based MgFe_2_O_4_ NPs and MgFe_2_O_4_@CaAlg NCs nanosensors is illustrated in Figure 8. In the case of the MgFe_2_O_4_ NPs nanosensor, the surface of MgFe_2_O_4_ NPs carriers a net negative charge, which is acquired by the symmetric stretching of oxygen atoms along the Fe^3+^–O^2−^ and Mg^2+^–O^2−^ bonds at the tetrahedral site and asymmetric stretching of Fe^3+^–O^2−^ and Mg^2+^–O^2−^ bonds at the octahedral site [50]. Since the N atom of MB exhibits a lower electronegativity compared to the O atom of the MgFe_2_O_4_ NPs. In this situation, the O atom carries a partial negative charge (δ−) and N carries a partial positive charge (δ+). As a result, dipole-dipole interactions might consequently originate from π–π interactions. Accordingly, nitrogen atoms of two MB molecules can interact with a single O atom from MgFe_2_O_4_ NPs.

In the case of the QCM-based MgFe_2_O_4_@CaAlg NCs nanosensor, the presence of alginate hydrogel increased the layer thickness of the MgFe_2_O_4_@CaAlg NCs nanosensor, causing more cavities to form. In addition, the presence of a polar side chain of carboxylic groups in the alginate hydrogel structure that contributed as functional groups with electron donors would have increased the density of negative charge and hydrophilicity of MgFe_2_O_4_@CaAlg NCs surface. Thus, the QCM-based MgFe_2_O_4_@CaAlg NCs nanosensor would interact more readily with MB through electrostatic interactions, besides the slight negative charge acquired by the MgFe_2_O_4_ NPs, which may help in further occurrence of π–π interactions. Moreover, the molecular structure of bio-adhesive alginate hydrogel that can absorb water at levels of more than 100 times its weight may influence the interaction of QCM-based MgFe_2_O_4_@CaAlg NCs nanosensor with MB molecules. Where the polar structure of alginate hydrogel enhanced the interactions with MB molecules that also have a polar structure causing more favorable adsorption. This interpreted why the adsorption capacities were higher for the MgFe_2_O_4_@CaAlg NCs nanosensor than for the MgFe_2_O_4_ NPs nanosensor.

### 3.4. Comparison of the QCM-Based MgFe_2_O_4_ NPs and MgFe_2_O_4_@CaAlg NCs Nanosensors Method with Other Methods in the Literature

The sensitive detection of the target analyte in the QCM sensor, surface-enhanced Raman scattering (SERS), and surface plasmon resonance (SPR) are crucial for the surface chemistry of the sensors. These techniques can be utilized to detect target analytes at low concentrations due to their rapid responses, high sensitivities, and lack of label requirement. The QCM-based MgFe_2_O_4_ NPs and MgFe_2_O_4_@CaAlg NCs nanosensor’s limit of detection for the MB was compared with it for SERS and SPR, suggesting that it might be utilized for real-time detection of MB at different concentrations. Additionally, the MB has also been identified using other methods such as ultraviolet-visible absorption spectrophotometry (UV-Vis), capillary electrophoresis (CE), fluorescence spectroscopy, and high-performance liquid chromatography (HPLC). The detection limit was provided by the QCM-based MgFe_2_O_4_ NPs and MgFe_2_O_4_@CaAlg NCs nanosensors compared to these techniques as summarized in Table 1.

Additionally, the nanocomposite-based sensors exhibit excellent room temperature selectivity, stability, and a fast response time behavior (around 5 min). The synergistic effect of QCM-based MgFe_2_O_4_ NPs and MgFe_2_O_4_@CaAlg NCs nanosensors is responsible for MB sensing enhancement. The potential application of the QCM-based MgFe_2_O_4_ NPs and MgFe_2_O_4_@CaAlg NCs nanosensors includes monitoring water pollution, particularly for toxic and hazardous MB dye. The MgFe_2_O_4_ NPs and MgFe_2_O_4_@CaAlg NCs nanosensors have been used to form substrates that were subjected to the QCM to achieve fast response times and high sensitivity at different temperatures. Responsivity and sensitivity were found to be dependent on MgFe_2_O_4_ NPs and MgFe_2_O_4_@CaAlg NCs size and density. The designed nanosensors demonstrated a response time of 5 min for the QCM-based MgFe_2_O_4_ NPs and MgFe_2_O_4_@CaAlg NCs nanosensors for MB under different temperatures and concentration operations. The results showed that the MgFe_2_O_4_ NPs and MgFe_2_O_4_@CaAlg NCs nanosensors may be regarded as a fast and effective method for MB dye detection in an aqueous solution utilizing the QCM technique. Consequently, the MgFe_2_O_4_ NPs sensing layer was upgraded with the CaAlg layer to decrease the response time and enhance the sensitivity, and the QCM sensor was developed for the detection and measurement of high concentrations of dye contaminant. Additionally, rather than waiting for adsorption equilibrium, it is feasible to specify a time when the mass of the developed QCM sensor starts to vary significantly to shorten the detection time. Table 2 represents the response time of different sensor systems from the literature compared with the achieved time by the QCM-based MgFe_2_O_4_ NPs and MgFe_2_O_4_@CaAlg NCs nanosensors.

## 4. Conclusions

In this work, novel QCM-based MgFe_2_O_4_ NPs and MgFe_2_O_4_@CaAlg NCs nanosensors have been developed for comparable detection of the high concentrations of MB in the water streams. The MgFe_2_O_4_ NPs were green synthesized using the extract of clove (*Syzygium aromaticum* L.). Then, the prepared MgFe_2_O_4_ NPs were coated using alginate hydrogel to obtain the MgFe_2_O_4_@CaAlg nanocomposite. The XRD results showed that MgFe_2_O_4_ NPs were formed in good crystallinity with minor differences observed for MgFe_2_O_4_@CaAlg NCs. The DLS and Zeta values revealed that the MgFe_2_O_4_ NPs and MgFe_2_O_4_@CaAlg possess a particles size distribution of 15 nm and 37 nm, and ζ-potentials of −32 mV and −4.8 mV, respectively. While the TEM images depicted that the MgFe_2_O_4_ NPs have a spherical shape and the MgFe_2_O_4_@CaAlg NCs were successfully coated with alginate hydrogel. Subsequently, the fabricated nanomaterials were employed as novel nanosensors based on the QCM method. The designed nanosensors were further used to monitor the highly concentrated MB of about 400 mg/L at different temperatures (25 °C, 35 °C, and 45 °C) and different initial MB concentrations (100 mg/L, 400 mg/L, and 800 mg/L). The results provided that as the temperature increases from 25 °C to 45 °C, the sensitivity of QCM-based MgFe_2_O_4_@CaAlg NCs for MB detection has improved. In addition, the QCM-based MgFe_2_O_4_@CaAlg NCs nanosensor exhibits a stable sensitivity towards different MB concentrations. On the contrary, the QCM-based MgFe_2_O_4_ NPs exhibited an opposing behavior for MB detection at higher both temperatures and concentrations of MB. Accordingly, it could be stated that the QCM-based MgFe_2_O_4_@CaAlg NCs is an efficient tool as a real-time rapid and sensitive nanosensor for MB detection in continuous-flow water streams.

## Figures and Tables

**Table 1:** Comparison of the QCM-based MgFe_2_O_4_ NPs and MgFe_2_O_4_@CaAlg NCs nanosensors method with other methods for detecting MB dye.

Method	LOD	Reference
Electrochemically synthesized SERS sensor based on gold and silver nanoparticles	9 × 10^−11^ M for e-AuNPs 5 × 10^−12^ M for e-AgNPs	[51]
SPR sensor using NiCo-Layered Double Hydroxide (NiCo-LDH)	0.005 mg/L	[52]
Micro-cloud point extraction and nonlinear laser wave-mixing detection interfaced with micellar capillary electrophoresis	81.6 pg/mL	[53]
Capillary electrophoresis	1.0 μg/mL	[54]
High-performance liquid chromatography	3 pmol	[55]
Solid-phase extraction (SPE) and ultra-performance liquid chromatography-tandem mass spectrometry (UPLC-MS/MS)	0.1 ng/mL	[56]
Fluorescence red-emitting CDs (CD-tetra)	10 nM	[57]
Electrochemical sensor consisting of amino-group-functionalized, multi-walled carbon nanotubes (NH_2_-*f*MWCNTs) immobilized on a glassy carbon electrode (GCE)	0.21 nM	[58]
UV–Vis spectrophotometry	0.65 μg/L	[59]
QCM-based MgFe_2_O_4_ NPs and MgFe_2_O_4_@CaAlg NCs nanosensors	400 mg/L (1.25× 10^−3^ M)	The current work

**Table 2:** Response sensitivity parameters of the different MB sensors based on sensing platforms in the literature compared with the QCM-based MgFe_2_O_4_ NPs and MgFe_2_O_4_@CaAlg NCs nanosensors.

Sensor	MB Concentration	Sensor Material Concentration	Operating Temperature (°C)	Response Time	Reference
Molecularly imprinted polymer-based QCM sensor (MIPs)	1–150 μg/L	1.5 mg	RT *	6000 s	[38]
Surface plasmon resonance sensor using NiCo-layered double hydroxide (SPR-glass/Au/NiCo-LDH)	0.005 mg/L	27.6 nm thick layer	RT	268 s	[52]
Electrochemical sensor consisting of amino-group-functionalized, multi-walled carbon nanotubes (NH_2_-*f*MWCNTs) immobilized on a glassy carbon electrode (GCE)	10 µM	0.89 mg/mL	RT	30 min	[58]
Molecularly imprinted fluorescence sensor based on the ZnO quantum dot core−shell structure (ZCF@MB-MIP)	0 to 100 μmol/L	37 mg/L	RT	15 min	[60]
Silver nanodecahedra (AgND)	10^−8^ to 10^−4^ M	0.5 mM	RT	15–50 min	[61]
Fe_3_O_4_@SiO_2_-GO microspheres based on SERS	1 × 10^−5^, to 1× 10^−7^ M	5 mg	RT	40 min	[62]
QCM-based MgFe_2_O_4_ NPs and MgFe_2_O_4_@CaAlg NCs nanosensors	400 mg/L (1.25× 10^−3^ M)	5 g/L	RT	5 min	The current work

* RT: Room Temperature.

## Data Availability

All the results and data used to support the findings of this study are included in the article.
